# Behavioral and Neuronal Investigations of Hypervigilance in Patients with Fibromyalgia Syndrome

**DOI:** 10.1371/journal.pone.0035068

**Published:** 2012-04-11

**Authors:** Laura Tiemann, Enrico Schulz, Andreas Winkelmann, Joram Ronel, Peter Henningsen, Markus Ploner

**Affiliations:** 1 Department of Neurology, Technische Universität München, Munich, Germany; 2 Department of Physical Medicine and Rehabilitation, Klinikum der Universität München, Munich, Germany; 3 Department of Psychosomatic Medicine and Psychotherapy, Technische Universität München, Munich, Germany; National Research & Technology Council, Argentina

## Abstract

Painful stimuli are of utmost behavioral relevance and thereby affect attentional resources. In health, variable effects of pain on attention have been observed, indicating alerting as well as distracting effects of pain. In the human brain, these effects are closely related to modulations of neuronal gamma oscillations. As hypervigilance as an abnormal increase of attention to external stimuli has been implicated in chronic pain states, we assumed both attentional performance and pain-induced gamma oscillations to be altered in patients with fibromyalgia syndrome (FMS). We recorded electroencephalography from healthy subjects (n = 22) and patients with FMS (n = 19) during an attention demanding visual reaction time task. In 50% of the trials we applied painful laser stimuli. The results of self-assessment questionnaires confirm that patients with FMS consider themselves hypervigilant towards pain as compared to healthy controls. However, the experimental findings indicate that the effects of painful stimuli on attentional performance and neuronal gamma oscillations do not differ between patients and healthy subjects. We further found a significant correlation between the pain-induced modulation of visual gamma oscillations and the pain-induced modulation of reaction times. This relationship did not differ between groups either. These findings confirm a close relationship between gamma oscillations and the variable attentional effects of pain, which appear to be comparable in health and disease. Thus, our results do not provide evidence for a behavioral or neuronal manifestation of hypervigilance in patients with FMS.

## Introduction

In health, pain fulfills a vital warning function to prevent physical harm. Due to this particular biological salience, painful stimuli involuntarily affect the allocation of attentional resources [1], [2]. On the one hand, prioritized processing of pain may affect the simultaneous processing of competing non-painful stimuli. Although somewhat controversial [3]–[5], there is substantial evidence that pain interferes with ongoing behavior by demanding the limited resources of selective attention ([6]–[9], for review see [1], [10], [11]). On the other hand, these distracting effects of pain are complemented by alerting effects [12], [13]. In line with these findings, several studies in healthy subjects observed variable effects of pain on behavioral performance, with painful stimulation yielding increased reaction times for some, as well as decreased reaction times for other subjects [14], [15]. In the human brain, these effects are closely related to modulations of neuronal gamma oscillations (30–100 Hz, [15], [16]).

In chronic pain states, pain does no longer fulfill a protective function, but represents a pathological condition with devastating effects on quality of life. Dysfunctional attentional processes have been implicated in the pathogenesis of chronic pain syndromes [1], [17]. In patients with fibromyalgia syndrome (FMS), hypervigilance as an abnormal increase of attention to external stimuli has been inferred from an increase in sensitivity to a large variety of painful [18]–[23] and non-painful stimuli [24]–[29]. Alternatively, but not mutually exclusively, a central augmentation of sensory input in terms of central sensitization [30] or deficient inhibitory control mechanisms [31], [32] may also account for the hypersensitivity in FMS. Experimentally, hypervigilance can be assessed using a behavioral paradigm inducing attentional interference [6]. Using such a primary task paradigm, evidence for behaviorally relevant hypervigilance in patients with chronic pain has been reported [33]. On the other hand, several experimental investigations failed to find experimental evidence for hypervigilance in patients with FMS [34], [35]. Thus, the existent literature is inconclusive, albeit not unsuggestive of hypervigilance in FMS. Accordingly, the role of hypervigilance in FMS remains to be elucidated.

We therefore recorded electroencephalography (EEG) during a visual attention task with concurrent painful stimulation to characterize both pain-related attentional performance and pain-induced neuronal gamma oscillations of patients with FMS and healthy subjects. Specifically, we aimed to address the following questions: (1) Do patients with FMS display a more pronounced pain-related distraction in an attention demanding paradigm as a behavioral correlate of hypervigilance towards pain? (2) Do patients with FMS and healthy subjects differ regarding visual and pain-induced gamma oscillations as a neuronal correlate of the attentional effects of pain? (3) Do patients with FMS and healthy subjects differ regarding the relationship between pain-induced effects on behavioral performance and pain-induced effects on neuronal gamma oscillations?

## Materials and Methods

### Subjects

The patient sample included data of 19 subjects (5 male, 14 female) with a mean age of 52 years (range 24–71 years). Patients were recruited from the Department of Physical Medicine and Rehabilitation, Klinikum der Universität München, and the Department of Psychosomatic Medicine and Psychotherapy, Technische Universität München. The inclusion criterion was fulfilling the American College of Rheumatology (ACR) criteria for FMS [36]. These require: (1) widespread pain involving both sides of the body, present above and below the waist as well as along the axial skeletal system, for at least three months. (2) Pain in 11 of 18 tender points on digital palpation. Exclusion criteria comprised neurologic, psychiatric, dermatological, or metabolic diseases, any disease causing chronic or acute pain other than FMS, and inability to interrupt therapy with centrally active analgesics (opioids) or coanalgesics (antidepressants, anticonvulsants) for a minimum of 7 days. The use of peripherally active rescue analgesics was allowed for up to 24 hours before study participation.

Additionally, 22 healthy subjects (2 male, 20 female) with a mean age of 47 years (range 25–66 years) participated in the study. Control and patient sample were matched for age (t = 1.4, p>0.1) and sex (χ^2^ = 2.1, p>0.1).

Written informed consent was obtained from all patients and healthy subjects before participation. The procedure was approved by the institutional review board (Ethikkommission der Technischen Universität München) and conducted in conformity with the declaration of Helsinki.

### Procedure

**Figure 1:**
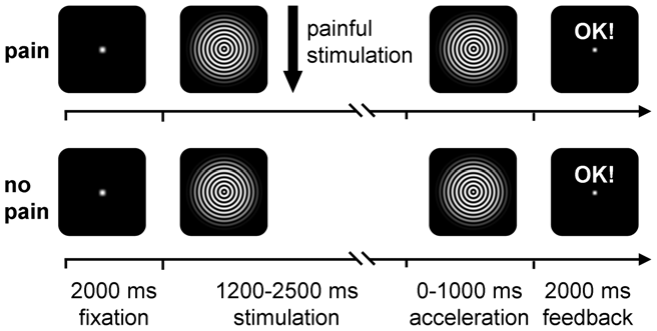
Paradigm. Subjects were presented a white fixation point against black background. After 2000 ms a circular sine wave grating contracting towards its center was shown. After a pseudorandomly varied duration of 1200 to 2500 ms the contraction accelerated, signaling the subjects to press a button with their right hand as fast as possible. Visual feedback followed the response. In 50% of the trials painful stimuli were applied to the left hand (*pain* trials). Apart from the application of painful stimuli, *pain* and *no pain* trials were identical. Time of painful stimuli was varied with respect to the onset of visual stimulation to avoid predictability of painful stimuli. Subjects were instructed to complete the visual task without becoming distracted by the painful stimulation.

Participants completed an attention-demanding visual reaction time task with interfering painful laser stimuli to investigate the effects of pain on attention (Fig. 1). The visual attention task is based on a well-established paradigm [15], [37] which reliably induces gamma oscillations in human visual cortices.

Subjects were presented a white fixation point against black background. After 2000 ms a circular sine wave grating contracting towards its center was shown. After a pseudorandomly varied duration of 1200 to 2500 ms the contraction accelerated, signaling the subjects to press a button with their right hand as fast as possible. Visual feedback followed the response. In 50% of the trials painful stimuli were applied to the left hand (*pain* trials). Apart from the application of painful stimuli, *pain* and *no pain* trials were identical. Time of painful stimuli was varied with respect to the onset of visual stimulation to avoid predictability of painful stimuli. Each subject completed one block consisting of 168 trials with a total duration of approximately 18 minutes. Subjects were instructed to complete the visual task without becoming distracted by the painful stimulation.

Prior to the EEG recordings, individual pain thresholds were determined using the method of limits. After the EEG recordings, subjects were asked to rate the mean pain intensity on a visual analogue scale. The scale ranged from 0 to 10, with 0 representing “no pain” and 10 representing “maximum tolerable pain”. The anchor descriptors *no pain*/*maximum tolerable pain* were chosen because they can be expected to yield a broader range of ratings in experimental studies in which low to moderate pain intensities are used, thereby yielding a higher sensitivity of ratings. In addition, we assessed pain vigilance and pain catastrophizing by using German versions of well-established questionnaires (Pain vigilance and awareness questionnaire, PVAQ [38]; Pain catastrophizing scale, PCS [39]).

### Stimuli

The task was performed on a personal computer with a 19 inch CRT monitor and a vertical refresh rate of 60 Hz using E-Prime software (Version 1.2, Psyc. Tools Inc.). Subjects were seated at a distance of approximately 70 cm from the computer screen.

Painful stimuli were delivered to the dorsum of the left hand using a Tm:YAG laser (Starmedtec GmbH, Starnberg, Germany) with a wavelength of 1960 nm, a pulse duration of 1 ms and a spot diameter of 5 mm. A distance pin mounted on the hand piece of the laser device ensured a constant distance between skin surface and laser device. Stimulation site was slightly varied after each stimulus. Stimulus intensity was individually adjusted to match a rating of 5 on a numerical rating scale ranging from 0 (“no pain”) to 10 (“maximum tolerable pain”). Subjects were exposed to white noise through headphones to cancel out noise of the laser device.

### EEG recordings

EEG data were recorded with an electrode cap (Easycap, Herrsching, Germany) and BrainAmp MR plus amplifiers (Brain Products, Munich, Germany) using the BrainVision Recorder software (Brain Products, Munich, Germany). Electrode montage included 64 electrodes consisting of the electrodes Fz/Cz/Pz, FP1/2, F3/4/7/8, C3/4, P3/4, T3/4/5/6 and O1/2 of the 10–20 system and the additional electrodes FPz, AFz, FCz, CPz, POz, Oz, Iz, AF3/4, F5/6, FC1/2/3/4/5/6, FT7/8/9/10, C1/2/5/6, CP1/2/3/4/5/6, P1/2/5/6, TP7/8/9/10, and PO3/4/7/8/9/10. Two more electrodes were fixed below the outer canthi of the eyes. The EEG was referenced to the FCz electrode, grounded at AFz, sampled at 1000 Hz and highpass-filtered at 0.1 Hz. The impedance was kept below 20 kΩ.

### Data analysis

#### Behavioral Data

To determine the effects of pain on visual task performance, reaction times were registered on a trial-by-trial basis. With regard to nerve conduction velocities, visual reaction times less than 150 ms were considered as false alarms and excluded from further analyses. Likewise, reaction times greater than 500 ms were considered as attentional errors and excluded from the analysis [40], [41]. This resulted in a total of 380 excluded trials (19%; *pain trials*: 222, *no pain trials*: 161). To compare the number of excluded trials between groups and conditions, we calculated a mixed model ANOVA with group as between- and condition as within-subject factor. The results do not show a significant interaction between group and condition (F_[1,39]_ = 0.3, p = 0.6), indicating that in the patient and the control group a comparable number of *pain*- and *no pain*-trials were excluded. For each subject, mean reaction times of *pain* and *no pain* trials were calculated and compared. Since reaction times [42] and neurophysiological responses [43] to painful stimuli are mainly observed between 100 and 500 ms after stimulus application, we expected painful stimuli which are applied shortly before a required response to interfere most profoundly with visual task performance. Analysis of behavioral data was therefore focused on *pain* trials where laser stimuli were applied during this time interval (200 or 500 ms before the acceleration, n = 24) and compared to otherwise identical *no pain* trials (n = 24).

#### Preprocessing of EEG data

EEG data were preprocessed using the BrainVision Analyzer software (Brain Products, Munich, Germany). Offline analysis included downsampling to 512 Hz, digital highpass filtering at 0.5 Hz and recomputation to the average reference. Downsampling included automatic lowpass filtering at 230 Hz. Independent component analysis was used to correct for vertical and horizontal eye movements. Trials with artifacts exceeding ±100 µV in any channel were automatically rejected.

#### Time-frequency analysis of EEG data

Since reaction times [42] and neurophysiological responses [43] to painful stimuli are mainly observed between 100 and 500 ms after stimulus application, analysis of behavioral responses focused on this interval. However, these trials are inevitably contaminated by motor activity related to the button press, which occurs shortly after the painful stimulation in these trials. We therefore focused the analysis of neurophysiological responses on *pain* trials where button presses occur at least 1800 or 2000 ms after the pain stimuli (n = 60). This ensures a long interval for the neurophysiological analysis, which is not contaminated by motor activity. It is important to note that the trials chosen for behavioral and neurophysiological analysis are identical except for the onset of acceleration of the moving visual stimulus. Since we were interested in the neuronal mechanisms *before* the acceleration, we assume both behavioral and neurophysiological trials to be identical concerning the pain-induced neuronal responses prior to the acceleration. The *pain* trials (n = 60) were compared to otherwise identical *no pain* trials (n = 60).

In order to transform the data from the time to the time-frequency domain, the complex demodulation procedure implemented in BESA 5.2 was used. Time-frequency transformation was performed for frequencies from 4 to 100 Hz in a time window from −1000 ms to 3500 ms with respect to the onset of visual stimulation. Frequencies were sampled in steps of 2 Hz, latencies in steps of 25 ms. Time-frequency representations were calculated as % signal change with respect to baseline. In the *no pain* condition, baseline was defined as −800 to −100 ms prior to stimulus onset. In the *pain* condition, trials had to be realigned to the laser stimuli that were applied either 500 or 700 ms after onset of the visual stimulation. Thus, the beginning of visual stimulation was preponed for 200 ms in 50% of the trials, and the baseline was adjusted accordingly. The time-frequency transformed data were averaged across trials for each condition and each electrode.

#### Source localization

The Multiple Source Beamformer Tool implemented in BESA was used to localize the cerebral sources of the visual and pain-induced gamma oscillations in each subject. Strongest gamma responses to visual stimuli were observed at latencies between 150 and 2500 ms and at frequencies between 48 and 54 Hz. Thus, localization was based on this time-frequency window and compared to a 1000 ms baseline. Strongest gamma responses to painful stimuli were observed at latencies between 75 and 200 ms after application of laser stimuli and at frequencies between 34 and 56 Hz. Thus, localization was based on this time-frequency window and compared to a 125 ms prestimulus baseline including visual activation. The resulting individual activation maps were averaged across subjects using BrainVoyager QX 1.9 (Brain Innovation, Maastricht, Netherlands).

#### Statistical analysis

Statistical analyses were performed using PASW (former SPSS) for windows (release 18, SPSS Inc., Chicago). Means between groups were compared using t-tests. Means between conditions and groups were compared using mixed-model analyses of variance (ANOVAs) with group as a between-subject factor and condition as a within-subject factor. Correlations were calculated using Spearman's rank correlation coefficient. Correlation coefficients were compared between groups by first converting each correlation coefficient into a z-score using Fisher's r-to-z transformation [44] and then calculating a z-score of the difference.

## Results

### Behavioral effects of pain

**Figure 2:**
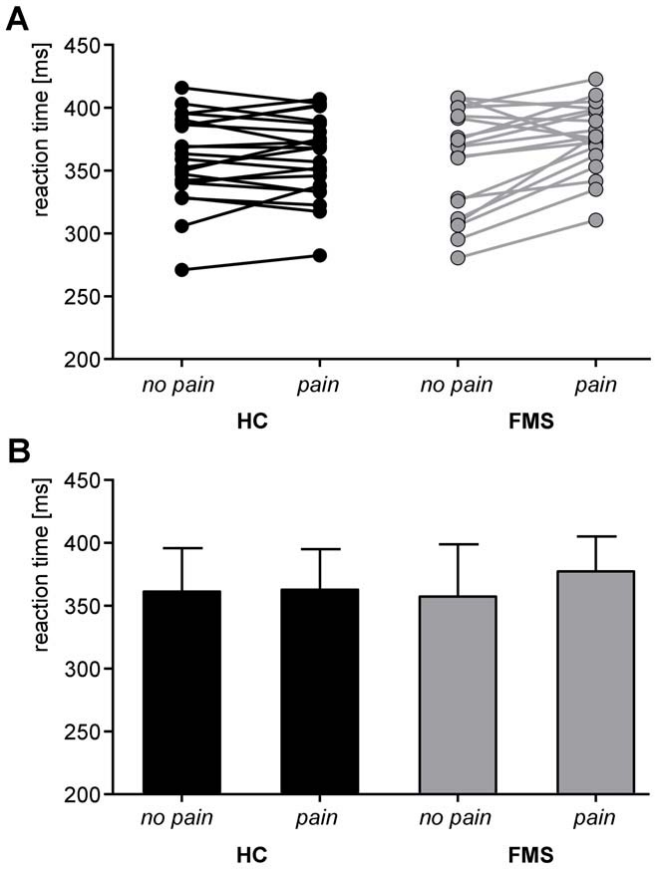
Behavioral results. ***A*** Single-subject reaction times for each group and condition. Please note that ascending lines connecting the *no pain* and *pain* conditions indicate a pain-induced prolongation of reaction times whereas descending lines indicate a reduction of reaction times. ***B*** Mean reaction times for each group and condition.

Mean objective stimulus intensity of painful laser stimuli across all participants was 528 mJ (SD = 86 mJ; range 320–700 mJ). These painful stimuli induced moderately painful sensations with a mean rating of 5.7 (SD = 1.8, range 2.0–9.4). Stimulus intensity (t = −1.08, p = 0.3) and pain ratings (t = 1.16, p = 0.3) did not differ between patients and healthy subjects. Though not statistically significant, a trend towards lower pain thresholds was noted in the patient group (t = −1.9, p = 0.07). Reaction times in the visual task served as a measure of visual attention. Mean reaction times across all trials were 361±33 ms (mean ± SD) for healthy subjects and 373±27 ms (mean ± SD) for patients with FMS. Reaction times across conditions did not differ between groups (t = 1.3, p = 0.2). Differences in reaction times between conditions served as measure of attentional interference. Painful stimuli did not homogeneously affect visual reaction times but yielded an increase of reaction times for some, as well as a decrease of reaction times for other participants (Fig. 2). In healthy subjects, these variable effects of pain on behavior have already been noted in previous studies on pain-cognition interactions ([14], [15], for review see [2]). In patients with FMS, we hypothesized a priori that the effects of pain on visual reaction times would be altered as an expression of disease-specific dysfunctional attentional processes. However, a two-way repeated measures ANOVA with one between subjects factor and one within subjects factor demonstrated no significant main effect of group (F_[1,39]_ = 1.73, p = 0.2) or condition (F_[1,39]_ = 1.98, p = 0.17). Moreover, the analysis did not reveal a significant condition x group interaction (F_[1,39]_ = 0.73, p = 0.4). Thus, healthy subjects and patients with FMS do not differ concerning the effects of pain on reaction time as a measure of attentional interference.

Interindividual differences in the effects of pain on visual reaction times were not correlated with pain threshold (r = −0.24, p = 0.13) or stimulus intensity (r = 0.06, p = 0.73). To control for differences in the individual attentional bias towards pain, we further considered the individual scores in the PVAQ as a measure of pain vigilance [1] and the PCS [45] as measure of pain catastrophizing. Patients with FMS considered themselves significantly more vigilant towards pain (t = 4.62, p<0.001) and showed a more pronounced tendency for catastrophizing (t = 4.53, p<0.01) compared to healthy subjects. However, the correlation between the effects of pain on attentional performance and pain vigilance/pain catastrophizing was not significant (r = 0.06, p = 0.71/r = −0.16, 0.30).

### Visual and pain-induced gamma oscillations

**Figure 3:**
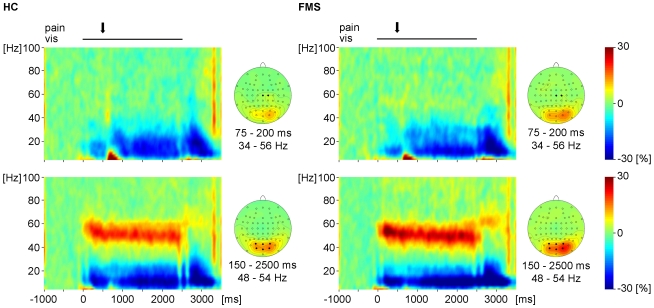
Visual and pain-induced gamma oscillations. The time-frequency-representations show group mean neuronal activity (% signal change) in *pain* trials used for neurophysiological analysis, averaged across central (Cz, C2) and occipital (POz, Oz, PO3/PO4, O1/O2) electrodes. Data are aligned to the onset of laser stimulation, which occurred 500 or 700 ms after onset of the visual stimulation, respectively. The topographic maps show the scalp distribution of gamma oscillations following visual (150–2500 ms after onset of visual stimulation, 48–54 Hz) and painful (575–700 ms after onset of visual stimulation, 75–200 ms after painful stimulation, 34–56 Hz) stimulation coded as % signal change as compared to baseline.

**Figure 4:**
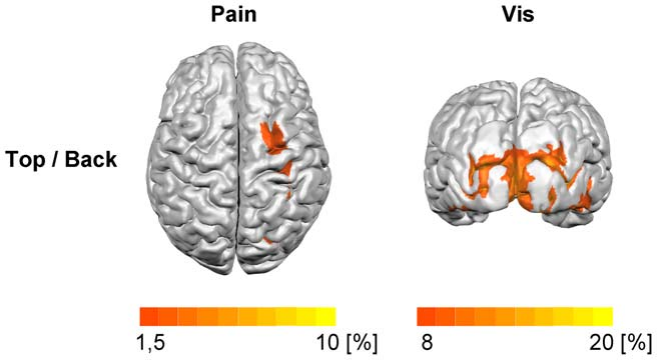
Source localization of visual and pain-induced gamma oscillations. Activations are maxima of mean activation maps superimposed on a normalized surface-rendered structural T1-weighted magnetic resonance image. Color-coded is the change of estimated activity in the target interval relative to the baseline in %.

Next, we investigated the effects of visual and painful stimuli on neuronal activity in the gamma frequency range. At occipital electrodes we found an increase in gamma oscillations which started about 150 ms after the onset of visual stimulation and lasted for the whole period of stimulus presentation (Fig. 3). Frequency of visual gamma oscillations varied interindividually between 40 and 60 Hz. The signal change was most prominent at electrodes POz, Oz, PO3/4 and O1/2. At these electrodes, gamma activity during visual stimulation (150 to 2500 ms, 48–54 Hz) was significantly increased compared to a prestimulus baseline (F_[1,39]_ = 45.2, p<0.001). The strength of visual gamma oscillations did not differ between healthy subjects and patients with FMS (F_[1,39]_ = 0.18, p = 0.7). At central electrodes, we found an increase in gamma oscillations between 75 and 200 ms after application of painful laser stimuli and at frequencies between 34 and 56 Hz (Fig. 3). The oscillations were most prominent at electrodes Cz and C2. At these electrodes, gamma activity was significantly increased after painful stimulation (75 to 200 ms, 34–56 Hz) compared to a prestimulus baseline (F_[1,39]_ = 7.9, p = 0.008). The strength of pain-induced gamma oscillations did not differ between healthy subjects and patients with FMS (F_[1,39]_ = 0.95, p = 0.34). Visual gamma oscillations were localized to the left and right occipital cortices (mean Talairach coordinates: left −25, −93, −4; right 27, −86, −7; Fig. 4). A maximum of pain-induced gamma oscillations was localized to the right primary somatosensory cortex (mean Talairach coordinates: 32, −20, 35; Fig. 4).

Thus, painful and visual stimuli can induce gamma oscillations in the somatosensory and visual system, respectively. Healthy subjects and patients with FMS do not differ concerning the strength of these visual and pain-induced gamma oscillations.

### Relationship between the behavioral and neuronal effects of pain in patients with FMS and healthy subjects

**Figure 5:**
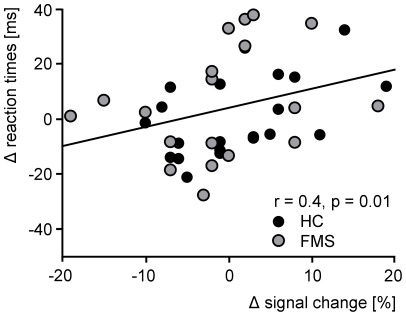
Relationship between pain-induced changes in visual gamma oscillations and pain-induced changes in visual task performance. Displayed is the correlation between the signal change of visual gamma oscillations (signal change_no pain_ – signal change_pain_) at occipital electrodes, and the change of reaction times after painful laser stimulation (RT_pain_ – RT_no pain_). Stronger pain-related modulations of visual gamma oscillations are associated with stronger pain-related modulations of reaction times.

Next, we investigated the behavioral relevance of neuronal gamma oscillations. If gamma oscillations are functionally relevant for attentional selection and enhanced processing of visual information, pain-induced changes in gamma oscillations would be correlated with pain-induced changes in behavior. We therefore correlated the effects of pain on visual reaction times with the effects of pain on visual gamma oscillations. The analysis revealed a significant positive correlation. Lower amplitudes of visual gamma oscillations after painful laser stimulation were associated with slower reaction times (r = 0.4, p = 0.01; Fig. 5), indicating a significant relationship between the pain-induced modulation of gamma oscillations in the human brain and the pain-induced modulation of attentional performance in a visual attention task.

We further investigated whether the correlation between the behavioral and neuronal effects of pain differed between healthy subjects and patients with FMS. We therefore compared the correlations after transforming the correlation coefficients from r to z-scores using Fisher's z transformation. The comparison revealed no significant difference of correlations between the control and patient group (z = −0.2, p = 0.84).

### Relationship between pain-induced and visual gamma oscillations in patients with FMS and healthy subjects

**Figure 6:**
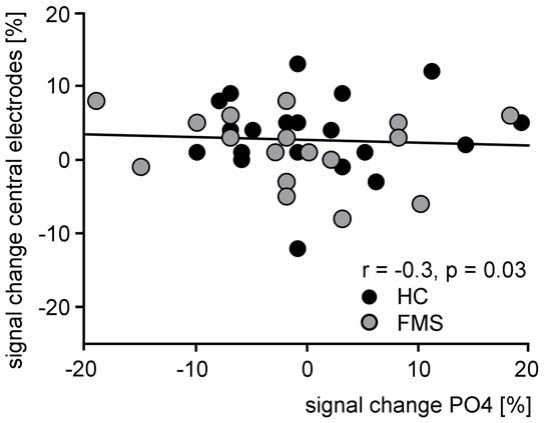
Relationship between pain-induced gamma oscillations and pain-induced changes in visual gamma oscillations. Displayed is the correlation between the signal change of pain-induced gamma oscillations measured 75–200 ms after painful laser stimulation at central electrodes and the signal change of visual gamma oscillations measured 200–350 ms after painful laser stimulation at occipital electrodes (signal change occipital_no pain_ – signal change occipital_pain_).

We were next interested whether pain-induced gamma oscillations in the somatosensory system affect visual gamma oscillations in the visual system. We therefore correlated pain-induced gamma oscillations at central electrodes (Cz, C2; 75–200 ms after painful stimulation) with pain-induced changes of visual gamma oscillations at occipital electrodes (Oz, POz, O1/2, PO3/4; 200–350 ms after painful stimulation). This correlation was not significant (r = −0.11, p = 0.5). However, previous studies indicated that visual gamma oscillations in the right, but not left, visual cortex are correlated with pain-induced gamma oscillations at central electrodes [15]. Moreover, visual gamma oscillations appeared to be right-lateralized (Fig. 3). Thus, we correlated pain-induced gamma oscillations at central electrodes (Cz, C2) with pain-induced changes of visual gamma oscillations at an electrode over the right occipital cortex (PO4). This analysis revealed a significant correlation (r = −0.34, p = 0.03; Fig. 6). Next, we were interested if this correlation between gamma oscillations in the somatosensory and visual system differed between healthy subjects and patients with FMS. The comparison revealed no significant difference of correlations between the control and patient group at occipital electrodes of both hemispheres (z = 0.3, p = 0.76) or the right hemisphere (z = 1.29, p = 0.2).

Thus, painful stimuli proportionally affect neuronal gamma oscillations in the somatosensory and visual system of the contralateral hemisphere. This relationship can likewise be observed in both healthy subjects and patients with FMS.

## Discussion

In the present study we investigated pain-related attentional interference and its neuronal correlates in patients with FMS. Referring to our initial questions, our results do not show a difference between healthy subjects and patients with FMS in (1) the effects of painful stimulation on reaction times, (2) the effects of painful stimulation on visual and pain-induced gamma oscillations, or (3) the relationship between pain-induced effects on behavioral performance and pain-induced effects on neuronal gamma oscillations. Thus, our findings do not show behavioral or neuronal effects of hypervigilance in patients with FMS.

### Visual and pain-induced gamma oscillations

Neuronal gamma oscillations have been related to attentional selection and enhanced processing of sensory information [46]–[48]. Here, we recorded pain-induced and visual gamma oscillations as neuronal correlates of visual and pain-related selective attention during a visual attention task with painful stimulation. To our knowledge, this is the first study to compare gamma oscillations as a neuronal signature of selective attention in healthy subjects and patients with FMS. However, healthy subjects and patients with FMS did not differ concerning their attentional performance or the strength of visual or pain-induced gamma oscillations. Due to the lack of a behavioral effect, however, it can not be excluded that gamma oscillations might represent a neuronal correlate of hypervigilance in situations where patients with FMS display behavioral signs of hypervigilance compared to healthy controls.

### Pain-related reallocation of visual and pain-induced gamma oscillations

In accordance with the hypothesis of limited attentional capacity, we expected simultaneous visual and painful stimulation to induce a reallocation of visual and pain-induced gamma oscillations in the human brain. Indeed, we observed a correlation between the strength of pain-induced gamma oscillations at central electrodes and the pain-induced modulation of visual gamma oscillations at occipital electrodes, indicating a proportional pain-related reallocation of gamma oscillations from visual to central areas. It has, however, to be noted that this correlation can not be interpreted as evidence for functional connectivity between these two areas. Still, the finding is in accordance with the results of a previous study using the same paradigm in a sample of healthy subjects [15]. In accordance with the hypothesis of dysfunctional attentional processes in FMS, either a disruption or a strengthening of the relationship between visual and pain-induced gamma oscillations would have been conceivable. However, a comparison of correlations between the patient and control group yielded no significant result, indicating that an inversely proportional reallocation of visual and pain-induced gamma oscillations can be likewise observed in healthy subjects and patients with FMS.

### Gamma oscillations as a neuronal correlate of the attentional effects of pain

In the present study we observed a significant correlation between the pain-induced effects on gamma oscillations and reaction times, respectively. This finding confirms a close relationship between gamma oscillations and reaction times as a measure of attentional performance [15], [49]–[52]. Here, we were particularly interested in whether the relationship between gamma oscillations and behavioral performance differed between patients with FMS and healthy subjects as an expression of pathologically altered attentional processing in FMS. However, we found the relationship between neuronal gamma oscillations and behavioral performance to be comparable in patients and healthy subjects.

### Hypervigilance in FMS does not affect behavioral performance or neuronal processing

The concept of hypervigilance in FMS gives reason to expect that patients are more easily distracted by painful stimulation and perform worse than healthy subjects in a behavioral paradigm inducing attentional interference. Our results confirm that patients consider themselves significantly more vigilant towards pain than healthy subjects. However, behavioral performance could not confirm an attentional bias towards pain in patients with FMS.

In health, variable effects of pain on behavior have been repeatedly described [14], [15]. This interindividual variability in the reaction to pain could not be attributed to differences in stimulus intensity or to differences in psychological factors [15]. Seminowicz and colleagues [14] proposed that the behavioral variability may relate to individual differences in the utilization of cognitive coping strategies. Moreover, it was suggested that differences in the balance of distracting and alerting effects of pain on attention may result in a behavioral continuum with varying grades from pain-induced *de*creased to pain-induced *in*creased performance [15]. In conformity with this hypothesis, dysfunctional attentional processes in FMS might correspond to a *shift* in the balance of distracting and alerting effects of pain towards the hypervigilant pole of this continuum. Here, however, we observed a comparable variability in the attentional effects of pain in the patient and the control group. Thus, our results do not show dysfunctional attentional processing of pain in patients with FMS.

Using self-assessment-questionnaires our results confirm that FMS patients consider themselves as hypervigilant towards pain which is in good agreement with the literature [27], [28], [53]. However, self-reported hypervigilance did not manifest itself in behavioral performance or neuronal processing. Likewise, the literature reports inconsistent results when hypervigilance in FMS is tested experimentally. On the one hand, at least one study reports evidence for behaviorally relevant attentional interference in patients with chronic pain [33]. In this study, the performance of chronic pain patients in an attention-demanding cognitive task was evaluated. The results showed that the disruption of attentional performance was most pronounced in patients with high pain intensity. However, the study sample comprised patients suffering from chronic pain of various origins and did not include a control group. Thus, the observed effects may not be specific to chronic pain in general or FMS in particular. On the other hand, several experimental investigations failed to find any evidence for behaviorally relevant hypervigilance in patients with FMS ([34], [35], for review see [54]). Whereas those studies evaluated the attentional bias regarding innocuous somatosensory [35] or threatening linguistic stimuli [34], the present study is the first to assess the pain-related attentional bias in FMS using a behavioral paradigm with concurrent *painful* stimulation, which is well suited to study pain-related attentional interference. Still, we did not find evidence for a behavioral or neuronal correlate of self-reported hypervigilance.

Decreased heat and pressure pain thresholds in FMS have been consistently reported by investigations using sensory testing methods [18]–[21], [23], [55]–[57]. In the present study, a tendency for lowered laser pain thresholds in the patient group could be noted. Considering the lack of a behavioral or neuronal correlate of hypervigilance in our study sample, one may speculate that these increases in pain sensitivity may not be due to hypervigilance for pain. Alternatively, but not mutually exclusively, central sensitization has been implicated in the pathophysiology of FMS (for review see [30]).

### Limitations

The generalizability of the present findings is limited in several aspects. First, behavioral studies have revealed that the capture of attention by pain is enhanced whenever pain is perceived as particularly threatening [1], [9]. Here, we did not control for the individual level of threat of the painful stimulation. Thus, it can not be precluded that patients with FMS are susceptible for pain-related attentional interference whenever the threat of painful stimulation is particularly high. Moreover, the clinical relevance of the experimental painful stimulation is debatable. The extent of attentional interference induced by brief, painful laser stimulation may not necessarily correspond to the extent of attentional interference induced by chronic pain experienced in FMS. Finally, it has been shown that the extent of pain-related distraction is related to the difficulty of the primary task [58]–[60]. Hence, it can not be excluded that the choice of paradigm accounts for the lack of behaviorally-relevant attentional interference in both patients and healthy controls. Conclusively it can be stated that the results obtained with the present paradigm and stimulation method do not necessarily generalize to other experimental or clinical settings.

### Conclusions

Using a behavioral paradigm to study attentional interference, we observed comparable task performance in patients and healthy controls. In both the patient and the control group, attentional effects of pain on behavioral performance were closely related to gamma oscillations in the human brain. However, since these effects did not differ between patients and healthy subjects, we could not confirm gamma oscillations as a neuronal correlate of perceived hypervigilance in FMS. The fact that perceived hypervigilance in patients with FMS does not manifest itself in behavioral performance and/or neuronal processing argues against a critical role of dysfunctional attentional processes in the pathogenesis of the disease. However, in order to conclusively assess the generalizability of the present findings, future studies taking into account the effect of stimulus qualities and paradigm characteristics are needed.
